# A Velocity Map Imaging Study of the Reactions of O^+^ (^4^S) With CH_4_

**DOI:** 10.3389/fchem.2019.00227

**Published:** 2019-04-12

**Authors:** Linsen Pei, James M. Farrar

**Affiliations:** Department of Chemistry, University of Rochester, Rochester, NY, United States

**Keywords:** ion-molecule, potential energy surface, atmospheric chemistry, methane, oxygen ion

## Abstract

We present a velocity map imaging study of the key ion-molecule reactions occurring in the O^+^(^4^S_3/2_) + CH_4_ (**X** ^1^A_1_) system at collision energies of 1.84 and 2.14 eV. In addition to charge transfer to form CH${}_{4}^{+}$ (**X** ^2^B_2_), we also present data on formation of CH${}_{3}^{+}$ (**X** ^1^A_1_'), for which the experimentally determined images provide clear confirmation that the products arise from dissociative charge transfer rather than hydride transfer. Experimental data are also presented on the formation of HCO^+^ through a transient [OCH_4_]^+^ complex living many rotational periods. Plausible reaction pathways and intermediate structures are presented to give insight into the routes for formation of these reaction products.

## Introduction

The reactions of the oxygen cation with methane and other small hydrocarbons at hyperthermal energies have received significant experimental and theoretical attention in the past 10 to 15 years. That interest has been motivated in large part by understanding the chemistry taking place in many environments, chief among them planetary atmospheres and interstellar clouds (Wakelam et al., 2012). The role that ions play in eroding the surfaces of spacecraft in low-earth orbit has also been a question addressed by these studies (Levandier et al., 2004). The relative simplicity of such reactions has stimulated theoretical studies that have led to fruitful comparisons with experiment, especially for the methane system, as exemplified by the pioneering experimental and theoretical study of the reactions in the O^+^(^4^S) + CH_4_ system by Levandier et al. (2004). Experimentally, this study employed the guided ion beam method to yield absolute cross sections as a function of collision energy for charge transfer, dissociative charge transfer and/or hydride abstraction, and carbon-oxygen bond formation. The overwhelmingly favored product for ground state O^+^ reactants was charge transfer to produce CH${}_{4}^{+}$. Theoretically, the study employed *ab initio* electronic structure calculations of quartet and doublet potential energy surfaces for the (O ∙ CH_4_)^+^ species that provided qualitative interpretations for the dominant reactive pathways.

More recent experiments by Cunha de Miranda et al. (2015) have focused on reactivity in the ground quartet (^4^S) and first two excited doublet (^2^D and ^2^P) states. Electronically state-selected reactant O^+^ ions were prepared by dissociative ionization of O_2_ using VUV photons from the DESIRS beamline. Like the experiments of Levandier et al., this study has employed guided beam methodology to determine absolute cross sections and branching ratios over an extended collision energy range from thermal to several eV. These experiments confirm that the dominant products for ground and excited state O^+^ reactants are CH${}_{4}^{+}$ and CH${}_{3}^{+}$, but with branching ratios that have a strong electronic state dependence. For ground state O^+^ reactants, the experimental data confirm the results of Levandier et al. that the CH${}_{4}^{+}$ charge transfer product is overwhelmingly favored. However, electronic excitation to the ^2^D and ^2^S states of O^+^ results in a strong preference for CH${}_{3}^{+}$ production. The authors did not report data for the products from C-O bond formation.

Both the experiments of Cunha de Miranda et al. and Levandier et al. make inferences about product energy and angular distributions from guided ion beam data taken at fixed collision energies. Under normal guided beam operation, the amplitude of the radiofrequency (rf) trapping voltage is sufficiently large to transmit all ions irrespective of the relative magnitudes of the longitudinal or transverse components of product velocities. In this mode of operation, measurements of the time-of-flight distributions for individual products yield projections of their speed distributions along the relative velocity vector. Additional experiments in which the rf amplitude is reduced significantly decrease the trapping well depth, allowing products with significant transverse velocities to escape detection. Such experiments yield more sensitive determinations of the forward and backward scattered product intensities, for which the latter are especially diagnostic of resonant charge transfer.

These experiments, along with computations that provide details of the potential energy surfaces, reactive intermediates and their structures, and related trajectory studies (Sun and Schatz, 2005) have answered a number of questions about the formation of the primary CH${}_{4}^{+}$ and CH${}_{3}^{+}$ products, as well as C-O bond formation, especially for ground state O^+^(^4^S). However, experimental limitations of the guided ion beam method have left open questions about whether CH${}_{3}^{+}$ is formed simply by dissociative charge transfer on the ground quartet state surface, or whether intersystem crossing to the doublet manifold facilitates hydride transfer to provide an additional route for CH${}_{3}^{+}$ production. In contrast to the ground quartet state of O^+^, each of the excited doublet states of O^+^ has a vacant 2*p* orbital that can accept an electron pair from the hydride ion H^−^. Furthermore, existing evidence showing that formation of the H_2_CO^+^ and HCO^+^ products occurs through a long-lived complex is suggestive, but not robust. In the present study, complete velocity space flux distributions obtained by the velocity map imaging (VMI) method provide the robust evidence necessary to clarify the mechanisms of these important reaction channels.

The objective of the experiments reported here is to clarify the mechanism by which CH${}_{3}^{+}$ is formed, and to provide deeper insight into the production of HCO^+^ by C-O bond formation. Velocity space images of these products, along with data for the charge transfer process, are offered in support of the claim that the methyl cation, CH${}_{3}^{+}$, is produced by dissociative charge transfer on the ground quartet surface via reaction (2) shown below, rather than hydride abstraction via reaction (7), and that the products H_2_CO^+^ and HCO^+^ appear to be formed by a spin-allowed condensation mechanism in the quartet manifold of potential surfaces. The observed reactions and their energetics are listed below:

(1) $${\text{O}}^{+}{(}^{4}{\text{S}}_{3/2})+{\text{CH}}_{4}(X^{1}{\text{A}}_{1})\to {\text{CH}}_{4}^{+}(X^{2}{\text{B}}_{2})+\text{O}{(}^{3}{\text{P}}_{2})\Delta \text{H}=–1.01\text{eV}$$

(2) $$\to {\text{CH}}_{3}^{+}(X^{1}{\text{A}}_{1^{\prime }})+\text{H}{(}^{2}{\text{S}}_{1/2})\text{+O}{(}^{3}{\text{P}}_{2})\Delta \text{H}=+0.75$$

(3) $$\to {\text{H}}_{2}{\text{CO}}^{+}(X^{2}{\text{B}}_{2})+2\text{H}{(}^{2}{\text{S}}_{1/2})\Delta \text{H}=–1.23$$

(4) $$\to {\text{H}}_{2}{\text{CO}}^{+}(X^{2}{\text{B}}_{2})+{\text{H}}_{2}(X^{1}\Sigma_{\text{g}}^{+})\Delta \text{H}=–5.68$$

(5) $$\to {\text{HCO}}^{+}(X^{1}\Sigma )+3\text{H}{(}^{2}{\text{S}}_{1/2})\Delta \text{H}=–0.08$$

(6) $$\to {\text{HCO}}^{+}(X^{1}\Sigma )+{\text{H}}_{2}(X^{1}\Sigma_{\text{g}}^{\text{+}})+\text{H}{(}^{2}{\text{S}}_{1/2})\Delta \text{H}=–4.53$$

(7) $$\to {\text{CH}}_{3}^{+}(X^{1}{\text{A}}_{1^{\prime }})+\text{OH}(X^{2}\Pi_{\text{i}})\Delta \text{H}=–3.69$$

Reaction energetics are taken from Table 1 of Sun and Schatz and Table 3 of Levandier et al.

## Experimental

As described previously (Pei and Farrar, 2012), the experiment is conducted with a crossed beam instrument employing velocity map product imaging (VMI) detection (Eppink and Parker, 1997). The imaging system determines all product velocities for a given mass in a single detection time window, yielding significant enhancement of detection efficiency through the intrinsic multiplex advantage of the method. Our implementation of VMI is based upon important developments from other laboratories (Reichert et al., 2000, 2002; Reichert and Weisshaar, 2002; Mikosch et al., 2006, 2008; Zhang et al., 2010).

The primary ion beam is formed by electron impact (Udseth et al., 1973) on a mixture of 10% CO in He. The primary product of electron impact on this mixture is He^+^, which then undergoes charge transfer with CO to form both parent and fragment C^+^ and O^+^ cations. The electronic state distribution of O^+^ produced in this manner can be assessed by using observations by Smith et al. (1992) which showed that charge transfer between O^+^ and CO is endoergic for ground state cations, but is exoergic for the ^2^S and ^2^D excited states. In a crossed beam geometry in which the O^+^ ion beam intersected a CO beam, we were unable to detect CO^+^ cations. This observation places a limit on excited state O^+^(^2^D, ^2^P) production of less than 1%.

In our experiment, the O^+^(^4^S) ions produced by electron impact are extracted, mass selected, and decelerated and focused by a series of ion optics, and the continuous beam of ions is delivered to the volume defined by the repeller and extraction electrodes of a VMI detector. The ion beam has a roughly triangular kinetic energy distribution with a FWHM of approximately 0.20 eV in the laboratory frame of reference.

The neutral beam is a supersonic expansion produced by a pulsed solenoid valve located 10 mm upstream from a 1 mm skimmer. The stagnation pressure of the CH_4_ gas behind the 0.1 mm diameter nozzle is 3 atm, and the resulting velocity distribution has a FWHM of ~6–8%. The pressure in the collision chamber is ~3 × 10^−7^ torr with the beams running.

Under the experimental conditions, the relative velocity of the reactants at a collision energy of 1.84 eV is 6,620 m/s. At E_col_ = 2.14 eV, the relative velocity of approaching reactants is 7,140 m/s.

The reactant beams intersect at the center of a collision volume defined by two circular electrodes of radius 38 mm spaced by 20 mm. The lower, repeller electrode and the upper, extractor electrode are held at ground potential as the ion and neutral beams intersect. Product detection is achieved by velocity map imaging (Eppink and Parker, 1997) with the two-electrode geometry described by the Suits research group (Townsend et al., 2003). Product detection is initiated by pulsed electric fields applied to the collision volume after reaction has taken place. The detection pulses, applied with separate high voltage pulse generators (DEI PVX-4140, 4150), are synchronized to the arrival of the central portion of the pulsed molecular beam and have a rise time and duration of 25 ns and 1 to 2 μs, respectively to allow all products to escape the volume between the repeller and extractor within the pulse duration.

Delayed pulsed extraction is achieved by pulsing the voltage on the repeller plate, V_1_, to +2300 V, the precise value dependent on transverse velocity and the filling factor for the MCP detector. The voltage V_2_ on the 13 mm aperture extraction electrode is pulsed to a value V_2_ = 0.65 V_1_. A grounded electrode with a 20 mm aperture placed 13 mm above the extraction electrode provides velocity mapping for the product ions at the imaging plane, located 0.6 m downstream from the grounded lens.

Prior to striking the imaging plane of the detector, defined by the front face of a pair of chevron-mounted microchannel plates, the ions pass through a grounded grid. The MCPs are gated by a pulse of base width 80 ns, which results in an effective “on” time of ~40 ns, during which the product ion cloud is recorded by the phosphor screen following the MCP anode. Under these operating conditions, the three-dimensional ion cloud is effectively “crushed” as it reaches the MCP detection plane.

The light image from the phosphor screen is recorded by a CCD camera (uEye 2230), which transfers the image via a USB interface to a lab computer controlled by LabView software. A typical image represents the accumulation of 5,000 to 20,000 repetitions of the pulsed valve.

## Results and Discussion

Velocity space images were collected for the CH${}_{4}^{+}$, CH${}_{3}^{+}$, and HCO^+^ products at collision energies 1.84 and 2.14 eV. Images for CH${}_{2}^{+}$ and H_2_CO^+^ formation were also detected. Because of the very strong similarities of these images to those for CH${}_{3}^{+}$ and HCO^+^, respectively, we have not shown them in this paper. Branching fractions for all five observed products are reported in Table 1 and are compared with the results of Cunha de Miranda et al.

**Table 1:** Product branching ratios.

	**E**_****rel****_ **=** **1.84 eV**		**E**_****rel****_ **=** **2.14 eV**	
**Product**	**Fraction**		**Fraction**	
CH${}_{4}^{+}$	0.44 ± 0.09^a^	(0.83)^b^	0.47 ± 0.09^a^	(0.83)^b^
CH${}_{3}^{+}$	0.49 ± 0.09^a^	(0.15)^b^	0.46 ± 0.09^a^	(0.15)^b^
CH${}_{2}^{+}$	0.06 ± 0.02^a^	(0.02)^b^	0.05 ± 0.02^a^	(0.02)^b^
H_2_CO^+^	0.005 ± 0.003^a^		0.005 ± 0.003^a^	
HCO^+^	0.008 ± 0.004^a^		0.01 ± 0.004^a^	

a This work.

b *Cunha de Miranda et al. (2015)*.

**Figure 1:**
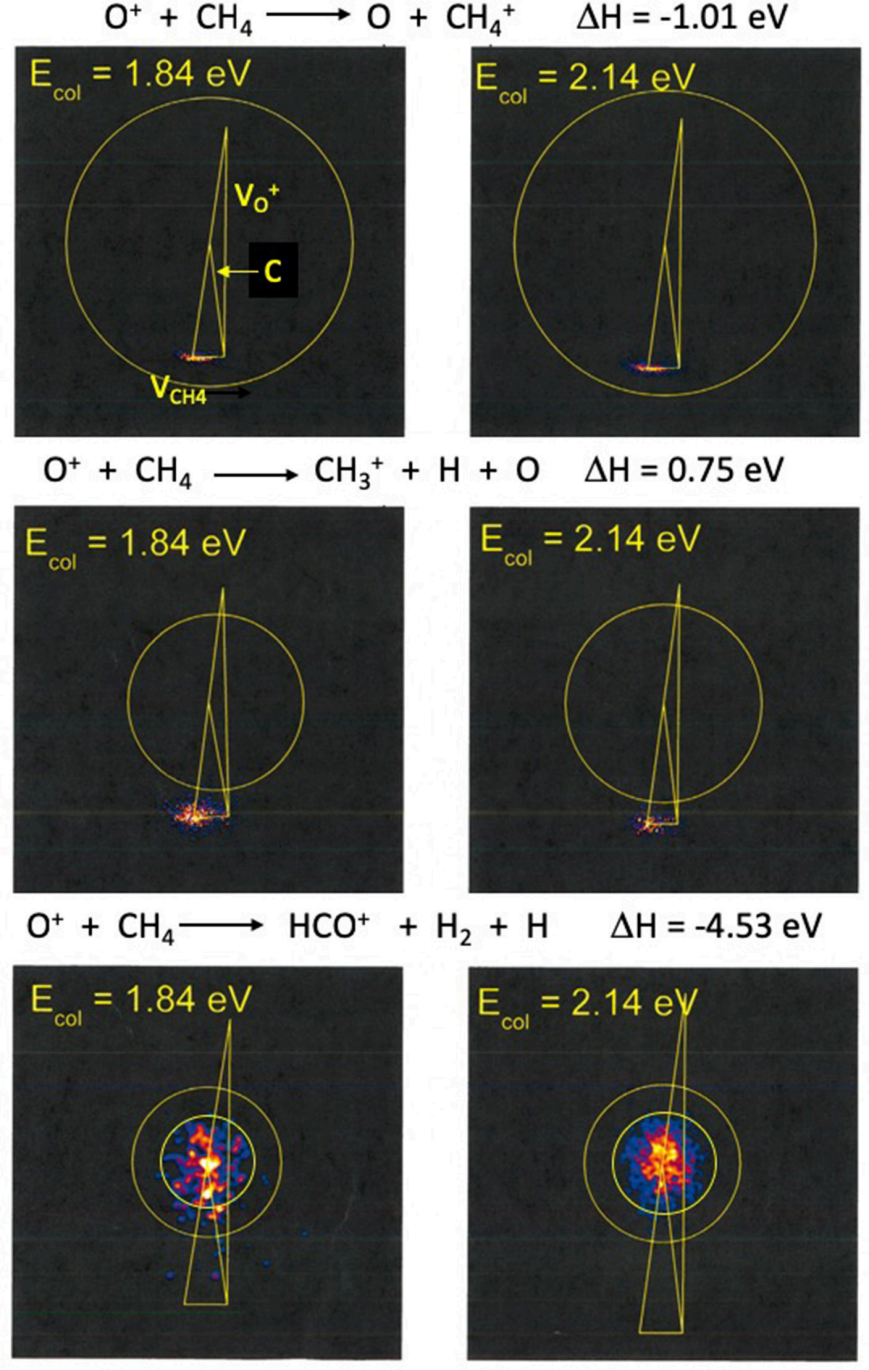
Ion images for CH${}_{4}^{+}$, CH${}_{3}^{+}$, and HCO^+^ production superimposed on the most probable Newton diagram. The directions of the O^+^ and CH_4_ beams are indicated on the top left image, and are not repeated on the other five frames to avoid cluttering the figure. The direction of the system center of mass is denoted by **C**. Laboratory velocities **v** are measured from the beam intersection point, and center of mass velocities **u** are measured relative to the tip of **C**. The left column of images corresponds to E_rel_ = 1.84 eV, and the right column corresponds to E_rel_ = 2.14 eV. Under the experimental conditions, the relative velocity of the reactants at a collision energy of 1.84 eV is 6,620 m/s. At E_col_ = 2.14 eV the relative velocity of approaching reactants is 7,140 m/s. The top row of images shows the formation of CH${}_{4}^{+}$ by charge transfer, and the circle corresponds to the barycentric speed associated with the thermochemical limit for reaction (1). The middle row of images shows CH${}_{3}^{+}$ produced by dissociative charge transfer, with the circle corresponding to the thermochemical limit for CH${}_{3}^{+}$ + O + H, the atomic products formed with zero relative kinetic energy, reaction (2). The bottom row of images shows HCO^+^ formation. The larger circle corresponds to maximum product speed allowed by energy conservation for the formation of HCO^+^ + H_2_ + H, reaction (6). The smaller circle corresponds to the maximum product speed allowed by energy conservation for the formation of HCO^+^ + H + H + H, reaction (5).

Figure 1 shows product images for charge transfer to form CH${}_{4}^{+}$, dissociative charge transfer and/or hydride transfer forming CH${}_{3}^{+}$, and C-O bond formation to form HCO^+^. The left column shows products formed at 1.84 eV and the right column shows the corresponding images at 2.14 eV. The dominant products correspond to CH${}_{4}^{+}$ and CH${}_{3}^{+}$ formation, with comparable intensities at both collision energies, as Table 1 shows. These results are consistent with those of Cunha de Marino et al., although the relative yield of CH${}_{3}^{+}$ is higher in our experiments. Secondary collisions occurring in the experiments with state-selected ions may be responsible for the differences in branching fractions. Images for H_2_CO^+^ production are very similar to those for HCO^+^ formation, and are not reported here.

The planar MCP detection system recovers images as product ion flux in Cartesian coordinates, either in the laboratory (v_x_, v_y_) or in the center of mass frame (u_x_, u_y_). Transformation of lab velocity (v_x_, v_y_) to center of mass velocity (u_x_, u_y_) is accomplished by a simple velocity shift **C** describing the motion of the center of mass of the collision system:

(8) $$\begin{array}{r} \text{u}=\text{v}-\text{C} \end{array}$$

Because the volume elements in both representations are equal, i.e., dv_x_ dv_y_ = du_x_ du_y_, (Wolfgang and Cross, 1969; Friedrich and Herman, 1984), center of mass flux P(u_x_, u_y_) may be visualized directly from the measured image. The full three-dimensional image and thus kinetic energy and angular distributions may be extracted from the experimental images by application of the BASEX algorithm (Dribinski et al., 2002). We do not report those distributions in this paper.

### CH${}_{4}^{+}$ Formation

At both collision energies, the CH${}_{4}^{+}$ charge transfer products, shown in the top panel of Figure 1, appear near the velocity of the incident CH_4_ reactant, consistent with energy resonance, with minimal conversion of reactant kinetic energy into product internal excitation. The circles shown on the images define the maximum kinetic energies accessible to the products, assuming that the total energy of the products, given as the sum of incident kinetic energy and reaction exoergicity, appears as product translation.

This strong peaking of product velocity is consistent with minimal deflection of the CH_4_ framework during electron transfer, and is consistent with large impact parameter collisions in which the electron is transferred to the ion at long range. The images show that the CH${}_{4}^{+}$ speed distributions are very narrow, indicative of a very small number of populated CH${}_{4}^{+}$ vibrational states. The energy widths of the CH${}_{4}^{+}$ product distributions are ~0.13 – 0.15 eV, or 1,000 to 1,200 cm^−1^.

From the perspective that charge transfer is driven not only by energy resonance, but also by the existence of favorable Franck-Condon (FC) factors between the reactant neutral and its corresponding ion, the narrow width suggests that the ionic potential energy surface is very steep in the FC region. However, the nature of the ionization process in CH_4_ requires more detailed consideration. Because the geometries of CH_4_ and CH${}_{4}^{+}$ are significantly different (Coulson and Strauss, 1962; Frost et al., 1967; Dixon, 1971), one expects that electron transfer, like photoabsorption, is constrained by the geometry change accompanying the process. Important early studies of charge transfer at thermal energies (Laudenslager et al., 1974) underscore the critical role of energy resonance and favorable Franck-Condon factors in determining reaction rates, but do not establish criteria that predict which effect is most important. In the present system, the charge transfer process requires crossings between potential surfaces that correspond asymptotically to (O^+^ + CH_4_) and (O + CH${}_{4}^{+}$), and the collision dynamics in the vicinity of these crossings modulate the effect of favorable Franck-Condon factors. The high dimensionality of the surfaces makes a detailed quantum analysis of those crossings difficult, if not intractable.

### CH${}_{3}^{+}$ Formation

The images for CH${}_{3}^{+}$ formation in the middle panels of Figure 1 clearly resemble those for direct charge transfer, centered on the tip of the CH_4_ neutral velocity vector. The images clearly lie outside the circles that define the locus of speeds consistent with three body collision-induced dissociation (CID). Those circles are defined by the maximum speed that a CH${}_{3}^{+}$ product would have when accompanied by an (O,H) pair bound with zero energy. The appearance of CH${}_{3}^{+}$ products outside those circles indicates that three-body CID is not the production mechanism for CH${}_{3}^{+}$. Rather, that product appears to originate from nascent CH${}_{4}^{+}$.

The energy resonance condition that governs the formation of CH${}_{4}^{+}$ imparts approximately 1.0 eV of internal energy to the ion. The kinetic energy contribution to the total energy available to the nascent CH${}_{4}^{+}$ arises from the initial kinetic energy less the kinetic energy removed by the oxygen atom also produced in the charge transfer process. Momentum conservation requires that the oxygen atom removes exactly half of the available kinetic energy, or ~0.9 to 1.1 eV. Thus, the total energy available to the nascent CH${}_{4}^{+}$ products of charge transfer ranges from 1.9 to a~2.1 eV. Reaction exoergicities show that the appearance potential for CH${}_{3}^{+}$ from CH${}_{4}^{+}$ is 1.84 eV. Considering the incident collision energy spread and the narrow but non-zero energy spread in the internal energy imparted to the nascent CH${}_{4}^{+}$ products, it is clear that a significant fraction of those products have sufficient energy to decay to CH${}_{3}^{+}$.

The methyl cation may also be formed by hydride transfer from CH_4_ to O^+^, a process exoergic by 3.69 eV. Previous studies have shown that hydride transfer from a methyl carbon atom to O^+^ (Curtis and Farrar, 1986) and to CH${}_{3}^{+}$ ions (Zabka et al., 1995) results in kinetic energy release distributions that are significantly broader and peaked to higher energies than electron transfer to the same approaching ion. Moreover, the ground quartet electronic state of O^+^ does not contain an empty 2*p* orbital to accommodate an electron pair from the hydride group H^−^. Therefore, hydride transfer to O^+^ should be strongly suppressed relative to electron transfer owing to its spin-forbidden character, and its dynamics should be very distinct from those of charge transfer. Thus, strong evidence supports the claim that CH${}_{3}^{+}$ is formed by spin-allowed dissociative charge transfer, reaction (2), rather than spin-forbidden hydride transfer, reaction (7). This claim has also been advanced by Cunha de Miranda et al.

The images for CH${}_{3}^{+}$ formation are broadened relative to their CH${}_{4}^{+}$ precursor images. The kinetic energy widths for the parent ions correspond to ~0.13 to 0.15 eV, and the widths of the CH${}_{3}^{+}$ daughter ions are ~0.06 eV larger. This broadening corresponds to roughly 20 to 30% of the total energy in excess of the dissociation threshold for CH${}_{4}^{+}$ decay to CH${}_{3}^{+}$, a result consistent with statistical unimolecular decay theories (Marcus, 1952).

### HCO^+^ Formation

The velocity space images for HCO^+^ formation are shown in the bottom row of images in Figure 1. Despite the kinematic constraint that the HCO^+^ products must appear close to the system center of mass in velocity space, the data are clear and precise, and allow the claim that HCO^+^ is distributed symmetrically in the center of mass frame and arises from decay of a long-lived precursor. The larger diameter circles shown in the images in the lowest row in Figure 1 correspond to the maximum speeds the HCO^+^ + H_2_ + H products could have if all available energy appears in translation. Similarly, the smaller circles correspond to the maximum speeds of HCO^+^ products formed along with three hydrogen atoms. At the lower collision energy, most of the flux falls within the smaller circle, suggesting that the spin-allowed pathway is the dominant one, and only a small fraction of the products are accompanied by molecular hydrogen formation. At the higher collision energy, all of the flux falls cleanly within the smaller circle.

**Figure 2:**
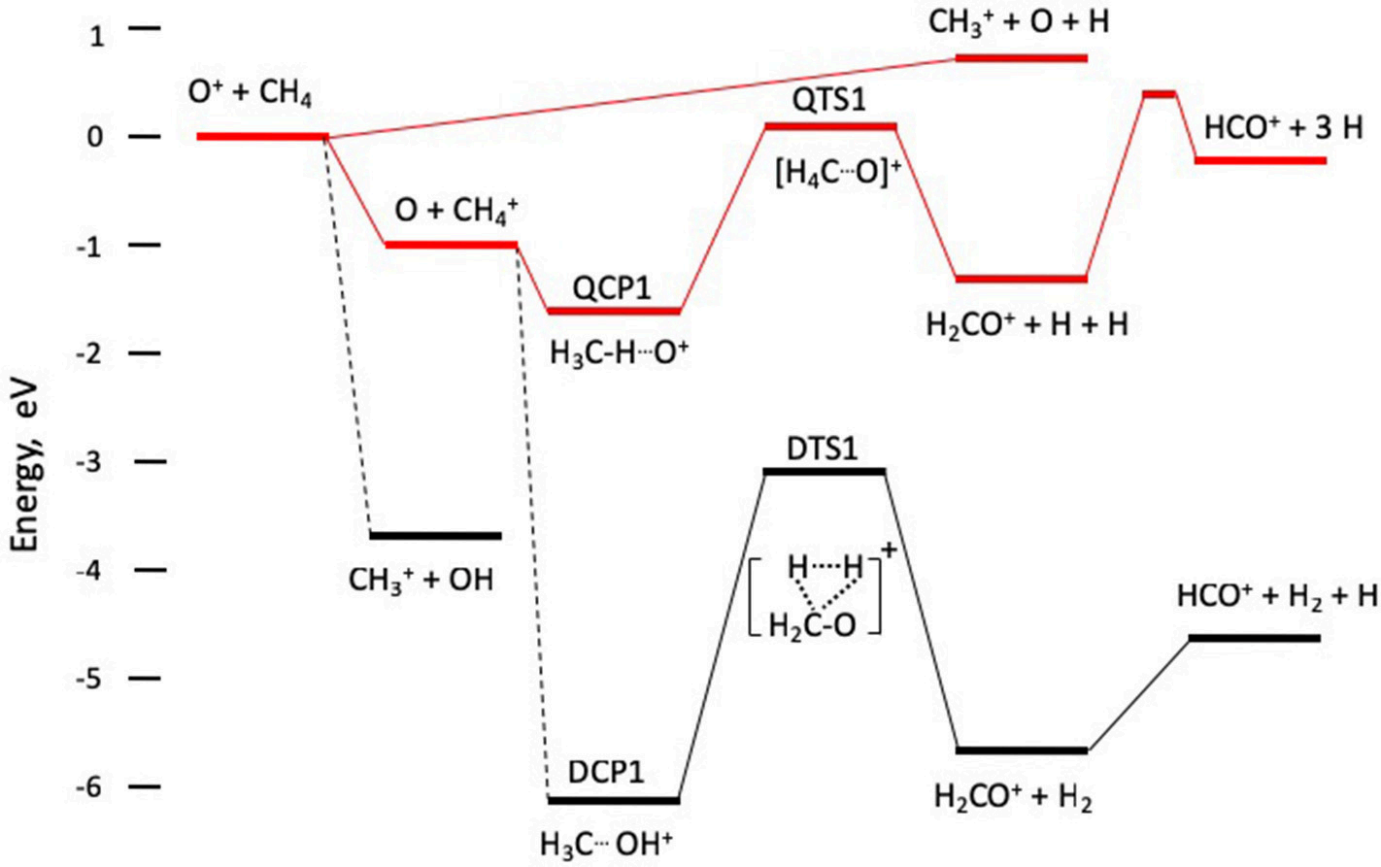
Schematic reaction coordinate diagram for formation of CH${}_{4}^{+}$, CH${}_{3}^{+}$, and HCO^+^ formation. The upper set of intermediates and transition states QCP1 and QTS1 evolving from O + CH${}_{4}^{+}$ to H_2_CO^+^ + H + H on the quartet potential surface, shown schematically in red. The lower set of intermediates and transition states DCP1 and DTS1 evolve from O + CH${}_{4}^{+}$ to H_2_CO^+^ + H_2_ on the doublet potential surface.

The computations reported by Levandier et al. (2004) that consider reaction on both quartet and doublet potential surfaces, and by Sun and Schatz (2005), who focus only on quartet surfaces, provide a computational basis for understanding the reaction dynamics. The reaction coordinate schematic shown in Figure 2 contains information extracted from those references. This pathway has been explored experimentally by Anderson et al. (Liu et al., 2005) and theoretically by Lorquet et al. (Pires et al., 1978), as well as in the work of Levandier et al. and Sun and Schatz.

As indicated in Figure 2, resonance charge transfer occurs on a quartet potential surface, and the nascent products O + CH${}_{4}^{+}$ lead to the important intermediate quartet complex denoted QCP1, as well as a doublet state complex labeled DCP1 in Levandier et al. and Sun and Schatz. In the complex QCP1, which lies 1.7 eV in energy below the O^+^ + CH_4_ reactants, a hydrogen atom from the methyl cation serves as a bridge between the carbon and oxygen atoms. The transition state QTS1 leading to the H_2_CO^+^ product has charge localized on the methane moiety, with significant spin density on the oxygen atom, and product cation is formed by ejection of two hydrogen atoms. This mechanism allows a doublet state ion to be formed in concert with two doublet state hydrogen atoms on a quartet state surface. The quartet surface shown in Figure 2 has fairly shallow wells (1.0 eV for the charge transfer products and 1.7. eV for QCP1), has a barrier at QTS1 that lies 0.07 eV above the approaching reactants, and products at 1.23 eV below the reactants. Given the relatively sparse densities of vibrational states in QCP1 and QTS1, it is unlikely that QCP1 will have a lifetime long enough to exhibit the signature of a long-lived complex.

The doublet state complex DCP1 is not directly accessible from the quartet state charge transfer products, and the dotted line connecting them, discussed by Levandier et al. (2004), suggests that an internal conversion process accesses motion on surfaces in the doublet manifold. The complex DCP1 is formed by insertion of O into a C-H bond, leading to the incipient C-O bond in H_2_CO^+^. Migration of a hydrogen atom from O to C leads to a three-center transition state, DTS1, lying ~3 eV above DCP1, that ejects molecular hydrogen in concert with the ground state formaldehyde cation, H_2_CO^+^. Formation of HCO^+^ over a barrier of ~0.6 eV is observed computationally, and Table 1 indicates that H_2_CO^+^ and HCO^+^ have comparable intensities.

The formation of H_2_CO^+^ via QCP1 on the quartet surface is accompanied by two hydrogen atoms. In contrast, H_2_CO^+^ production via DCP1 on the doublet surface is accompanied by molecular H_2_ formation. Thus, the total energies available to the quartet state products, the sum of collision energy and exoergicity for HCO^+^ + 3H, are ~1.9 and 2.2 eV for the two collision energies respectively, while ~6.4 and 6.7 eV are accessible to the HCO^+^ + H_2_ + H products formed on the doublet surface. At the lower (higher) collision energy, products formed with more than 1.9 (2.2) eV of translation must be assigned to formation on the doublet surface, while products formed with 1.9 (2.2) eV or less may be formed on either surface. Qualitative bounds on the kinetic energy release can be gleaned from the images in Figure 1, particularly at the higher collision energy. The data show clearly that kinetic energy release is fairly small, with most of the flux confined within the smaller of the two circles shown in each of the images in the bottom panel of Figure 1. Those smaller circles correspond to the maximum speeds accessible to HCO^+^ + 3H products. The images support the claim that a very small fraction of the products are formed following internal conversion to the doublet surface.

## Conclusions

The experimental data reported here for CH${}_{4}^{+}$, CH${}_{3}^{+}$, and HCO^+^ formed in the reactions of O^+^(^4^S) with CH_4_, in conjunction with reaction pathways proposed by Levandier et al. and Sun and Schatz, provide clear evidence that the primary CH${}_{4}^{+}$ product is formed in a very narrow range of vibrational states by resonant charge transfer. The experimental data for CH${}_{3}^{+}$ formation provide strong evidence that this product, formed with comparable cross section, arises from dissociation of nascent CH${}_{4}^{+}$ on the quartet potential surface, rather than by the spin-forbidden process of hydride transfer.

Although HCO^+^ production is a minor channel, formed at less than 1% of the total product yield, the pathways for its formation are quite interesting. The spin-allowed quartet surface pathway leads to formation of H_2_CO^+^ in conjunction with ejection of two hydrogen atoms. Unimolecular decay of the nascent H_2_CO^+^ product by C-H bond cleavage yields HCO^+^ + H with ~60 to 65% yield. Computations also suggest a viable pathway for H_2_CO^+^ production (with subsequent decay to HCO^+^) in concert with an H_2_ molecule via internal conversion from the lowest quartet state to the doublet manifold.

The experimental data show that the kinetic energy release for H_2_CO^+^ formation and subsequent decay to HCO^+^ is quite low, ~2 eV or smaller. No products are formed with kinetic energies in the range between 2.2 and 6.7 eV, where energy conservation constrains products to be formed on the doublet surface. There is no experimental evidence to claim that any of the observed reaction products must be formed by spin-forbidden processes. Although one might expect that the tight transition state DTS1 preceding H_2_ ejection would produce translationally excited products, the data provide no evidence for that expectation.

The experimental data presented here have demonstrated that ion imaging has yielded additional insights into the benchmark O^+^ + CH_4_ system, complementing important contributions, both experimental and theoretical, already in the literature. We hope that imaging methods will continue to play an important role in elucidating the dynamics of prototypical systems in atmospheric chemistry and astrochemistry.

## Data Availability

The datasets generated for this study are available on request to the corresponding author.

## Author Contributions

LP conducted the experiment reported here, analyzed the data, and produced the figures. JF. suggested the problem and wrote up the initial draft of the presentation of the results. LP and JF discussed the analysis and interpretation of the experimental results together.

### Conflict of Interest Statement

The authors declare that the research was conducted in the absence of any commercial or financial relationships that could be interpreted as a potential conflict of interest.
